# Establishment of an Ultrasound Malignancy Risk Stratification Model for Thyroid Nodules Larger Than 4 cm

**DOI:** 10.3389/fonc.2021.592927

**Published:** 2021-06-29

**Authors:** Xuehua Xi, Ying Wang, Luying Gao, Yuxin Jiang, Zhiyong Liang, Xinyu Ren, Qing Gao, Xingjian Lai, Xiao Yang, Shenling Zhu, Ruina Zhao, Xiaoyan Zhang, Bo Zhang

**Affiliations:** ^1^ Department of Ultrasound, China-Japan Friendship Hospital, Beijing, China; ^2^ Department of Ultrasound, Peking Union Medical College Hospital, Beijing, China; ^3^ Department of Pathology, Peking Union Medical College Hospital, Beijing, China

**Keywords:** thyroid nodules, thyroid cancer, ultrasound, risk stratification, size

## Abstract

**Background:**

The incidence and mortality of thyroid cancer, including thyroid nodules > 4 cm, have been increasing in recent years. The current evaluation methods are based mostly on studies of patients with thyroid nodules < 4 cm. The aim of the current study was to establish a risk stratification model to predict risk of malignancy in thyroid nodules > 4 cm.

**Methods:**

A total of 279 thyroid nodules > 4 cm in 267 patients were retrospectively analyzed. Nodules were randomly assigned to a training dataset (n = 140) and a validation dataset (n = 139). Multivariable logistic regression analysis was applied to establish a nomogram. The risk stratification of thyroid nodules > 4 cm was established according to the nomogram. The diagnostic performance of the model was evaluated and compared with the American College Radiology Thyroid Imaging Reporting and Data System (ACR TI-RADS), Kwak TI-RADS and 2015 ATA guidelines using the area under the receiver operating characteristic curve (AUC).

**Results:**

The analysis included 279 nodules (267 patients, 50.6 ± 13.2 years): 229 were benign and 50 were malignant. Multivariate regression revealed microcalcification, solid mass, ill-defined border and hypoechogenicity as independent risk factors. Based on the four factors, a risk stratified clinical model was developed for evaluating nodules > 4 cm, which includes three categories: high risk (risk value = 0.8-0.9, with more than 3 factors), intermediate risk (risk value = 0.3-0.7, with 2 factors or microcalcification) and low risk (risk value = 0.1-0.2, with 1 factor except microcalcification). In the validation dataset, the malignancy rate of thyroid nodules > 4 cm that were classified as high risk was 88.9%; as intermediate risk, 35.7%; and as low risk, 6.9%. The new model showed greater AUC than ACR TI-RADS (0.897 *vs.* 0.855, p = 0.040), but similar sensitivity (61.9% *vs.* 57.1%, p = 0.480) and specificity (91.5% *vs.* 93.2%, p = 0.680).

**Conclusion:**

Microcalcification, solid mass, ill-defined border and hypoechogenicity on ultrasound may be signs of malignancy in thyroid nodules > 4 cm. A risk stratification model for nodules > 4 cm may show better diagnostic performance than ACR TI-RADS, which may lead to better preoperative decision-making.

## Introduction

Thyroid nodules occur in up to 68% of people in the general population worldwide, and 5-15% of nodules are malignant (1, 2). Research shows that the incidence and mortality of thyroid cancer, including thyroid nodules > 4 cm, has been on the rise in recent years and warrants further research (3). Both the 2017 Thyroid Cancer Staging Manual of the American Joint Committee on Cancer (AJCC) and the 2015 Management Guidelines of the American Thyroid Association (ATA) (Referred to as ATA) for adult patients with thyroid nodules and differentiated thyroid cancer list thyroid nodules > 4 cm as an important factor for surgical decision-making, as integrated into the Tumor, Node, Metastasis (TNM) staging system (4, 5). Recent guidelines have suggested ultrasound risk stratification patterns to assess the malignant risk of thyroid nodules, including the American College Radiology Thyroid Imaging Reporting and Data System (ACR TI-RADS) (4, 6–9). However, these methods are based mostly on research of thyroid nodules < 4 cm. For example, fine-needle aspiration biopsy (FNAB) is considered to be the gold standard for preoperative diagnosis of thyroid cancer. However, FNAB shows lower sensitivity and higher rates of false negative results in the case of thyroid nodules > 4 cm (10–12). Decisions related to surgery and other treatments may be affected if pre-operative assessment of thyroid nodules is inaccurate or incomplete. Thus, distinguishing malignant from benign nodules pre-operatively would assist in diagnosis and decision-making. The aim of the current study was to identify factors that predict malignancy in thyroid nodules > 4 cm and construct an applicable risk stratification model.

## Material and Methods

**Figure 1:**
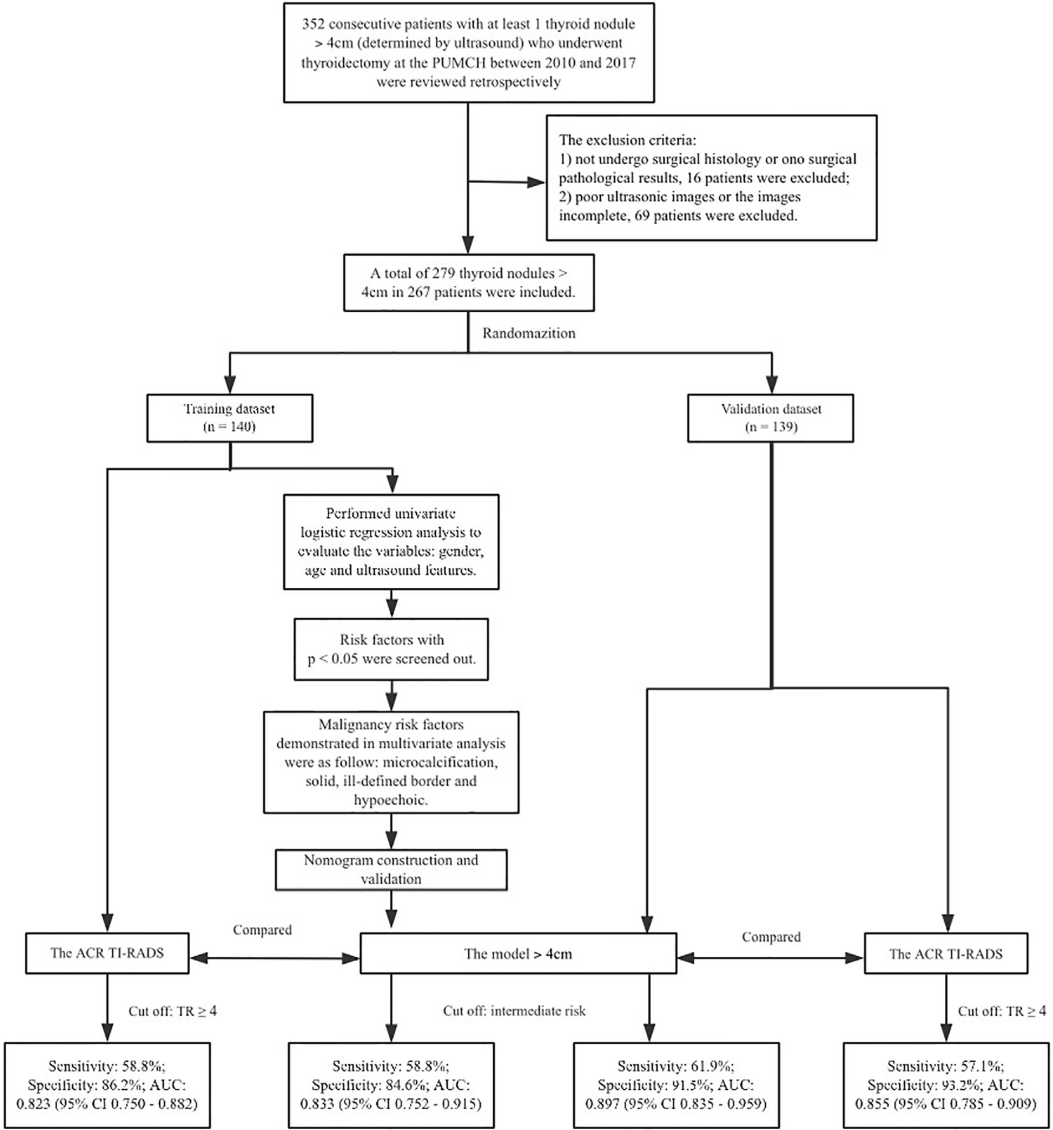
The study protocol.

Study protocols are shown in **Figure 1**.

### Patients

Consecutive patients with at least one thyroid nodule > 4 cm who underwent thyroidectomy at the Peking Union Medical College Hospital (Beijing, China) between 2010 and 2017 were reviewed retrospectively. The inclusion criteria were as follows :(1) the size of the nodule was > 4 cm in its longest diameter, as determined by ultrasonography; and (2) the nodule had not previously been treated surgically. The exclusion criteria were: (1) pathology results from surgical tissue were unavailable for the patient, or (2) ultrasound images were poor or incomplete. A total of 279 thyroid nodules in 267 patients were included for the study (**Figure 1**). The patients comprised 185 females aged 60.0 ± 13.2 yr and 82 men aged 49.9 ± 13.3 years (**Figure 1**).

The study was approved by the Institutional Review Board of Peking Union Medical College Hospital. All patients provided informed consent for their clinical data to be published anonymously for research purposes.

### Ultrasound Examination

Relevant clinical and ultrasound data of all cases were extracted from the central hospital database. Ultrasound examinations were performed with Phillips IU 22, GE Logiq 9 or GE Logiq 7 devices equipped with a linear array probe of 8-15 MHz A convex array probe of 5-12 MHz was used for larger thyroid nodules. Ultrasound images were retrospectively reviewed by two radiologists who had more than 5 years’ experience analyzing thyroid ultrasound, and who were blinded to patients’ clinical and pathological results. The age and sex of the patients were recorded, as were ultrasound features of each nodule, including size, composition, echogenicity, margin, shape, border, calcification and halo. The two radiologists resolved any inconsistencies in their reviews through discussion.

All thyroid nodules were also evaluated using ACR TI-RADS, Kwak TI-RADS and ATA (4, 6, 9). According to the ACR TI-RADS, points were given for all ultrasound features in a nodule. Features suggesting malignancy were awarded additional points. The total points determined the nodule’s ACR TI-RADS level, which ranged from TR1 (benign) to TR5 (high probability of malignancy) (**Table 1**).

**Table 1:** Point system in ACR TI-RADS (6).

Points	Categories				
	Composition	Echogenicity	Shape	Margin	Echogenic foci
0	cystic or almost completely cystic; spongiform	anechoic	wider-than-tall	smooth;ill-defined	none;large comet-tail artifacts
1	mixed cystic and solid	Hyperechoic;isoechoic			macrocalcifications
2	solid or almost completely solid	hypoechoic		lobulated or irregular	peripheral (rim) calcifications
3		very hypoechoic	taller-than-wide	extra-thyroidal extension	punctate echogenic foci

Points across the five categories are summed to determine the malignancy probability level: TR1 (benign, 0 point), TR2 (not suspicious, 2 points), TR3 (mildly suspicious, 3 points), TR4 (moderately suspicious, 4-6 points), and TR5 (highly suspicious, 7 points or more).

### Statistical Analysis

Data analysis was performed using SPSS 19.0 (IBM, Chicago, IL, USA) and p < 0.05 as the definition of statistical significance. Nodules were randomly assigned to a training dataset or validation dataset (13). Continuous data were reported as means ± SD, and inter-group differences were assessed for significance using Student’s *t*-test. Differences in categorical data were assessed using the χ^2^-test or Fisher’s exact test as appropriate. Categorical variables were classified based on clinical and ultrasound findings. The continuous variable age was transformed into a categorical variable (≥55 or < 55 years) based on a previous report (5).

The variables that were identified as statistically significant prognostic factors were assessed in multivariate logistic regression analysis. A nomogram was constructed based on the results of multivariate analysis and validated using the validation dataset, using the *rms* package in R 3.6.0. The diagnostic performance of the nomogram was evaluated using the concordance index (C-index) and area under the receiver operating characteristic curve (AUC). Bootstrapping validation (1,000 bootstrap resamples) was used to calculate a relative corrected C-index (14). A calibration curve (1,000 bootstrap resamples) was generated to verify the calibration of the prediction nomogram.

A model for risk stratification of thyroid nodules > 4 cm was established according to the nomogram. Nodules were classified as high, intermediate, or low risk. The cut-off values for the three-level risk stratification were determined according to AUC, sensitivity, specificity, positive predictive value (PPV), and negative predictive value (NPV). Accuracy was calculated according to the cut-off value.

Similarly, the diagnostic performance of the ACR TI-RADS was evaluated in terms of AUC, sensitivity, specificity, PPV, NPV and accuracy. The results were compared between this reference standard and proposed model.

## Results

### Clinical Characteristics of the Sample

The study comprised 279 nodules in 267 patients, including 50 (17.9%) malignant and 229 (82.1%) benign nodules (**Table 2**). Of the 267 patients with a mean age of 50.6 ± 13.2 years (range, 17-80 years), 185 were women (60.0 ± 13.2 years) and 82 were men (49.9 ± 13.3 years).

**Table 2:** Ultrasound features and ACR TI-RADS levels for 279 thyroid nodules > 4 cm.

Feature or level	All nodules (n = 279)	Training dataset (n = 140)			Validation dataset (n = 139)		
		Pathological diagnosis		p-value	Pathological diagnosis		p-value
		Malignant (n = 29)	Benign (n = 111)		Malignant (n = 21)	Benign (n = 118)	
Ultrasound features							
Size (cm)	5.24 ± 1.1	5.7 ± 1.5	5.1 ± 1.0	0.349	5.0 ± 0.5	5.3 ± 1.2	
Composition				<0.001			<0.001
Solid	64 (22.9)	15 (51.7)	13 (11.7)		14 (66.7)	22 (18.6)	
Mixed cystic and solid/Cystic	215 (77.1)	14 (48.3)	98 (88.3)		7 (33.3)	96 (81.4)	
Echogenicity				0.001			<0.001
Hypoechoic	122 (43.7)	22 (75.9)	44 (39.6)		17 (81.0)	39 (33.1)	
Other*	157 (56.3)	7 (24.1)	76 (60.4)		4 (19.0)	79 (66.9)	
Border				<0.001			<0.001
Ill-defined	28 (10)	9 (31.0)	6 (5.4)		8 (4.2)	5 (4.2)	
Defined	251 (90)	20 (69.0)	105 (94.6)		13 (61.9)	113 (95.8)	
Margin				<0.001			<0.001
Irregular/lobulated	42 (15.1)	10 (34.5)	7 (6.3)		10 (47.6)	15 (12.7)	
Smooth	237 (84.9)	19 (65.5)	104 (93.7)		11 (52.4)	103 (87.3)	
Shape				–			–
Taller-than-wide >1	0 (-)	0 (-)	0 (-)		0 (-)	0 (-)	
≤1	279 (100)	29 (100)	111 (-)		29 (100)	111 (-)	
Calcification				<0.001			<0.001
Microcalcification	21 (7.5)	9 (31)	3 (2.7)		8 (38.1)	1 (0.8)	
Others^#^	258 (92.5)	20 (69)	108 (97.3)		13 (61.9)	117 (99.2)	
Halo				0.014			0.185
Absent	127 (45.5)	17 (58.6)	43 (38.7)		12 (57.1)	55 (46.6)	
Irregular	50 (17.9)	8 (27.6)	20 (18.0)		5 (23.8)	17 (14.4)	
Regular and thin	102 (36.5)	4 (13.8)	48 (43.2)		4 (19.0)	46 (39.0)	
ACR TI-RADS				<0.001			<0.001
TR5	16 (5.7)	8 (27.6)	1 (0.9)		6 (28.6)	1 (0.8)	
TR4	35 (12.5)	10 (34.5)	7 (6.3)		7 (33.3)	11 (9.3)	
TR3	150 (53.8)	10 (34.5)	68 (61.3)		8 (38.1)	64 (54.2)	
TR2	73 (26.2)	1 (3.4)	34 (30.6)		0 (-)	38 (32.2)	
TR1	5 (1.8)	0 (-)	1 (0.9)		0 (-)	4 (3.4)	

Values are mean ± SD or n (%), unless otherwise noted. *Including isoechoic, hyperechoic, and anechoic. ^#^Including macrocalcification, large comet-tail artifacts and none. ACR, American College Radiology; TI-RADS, Thyroid Imaging Reporting and Data System.

Nodules were randomly assigned to a training dataset (n=140) or validation dataset (n=139) (**Table 2**). There was no significant difference in the ultrasound features between the two datasets. In this study, 264 (94.6%) thyroid nodules were performed with Phillips IU 22, 12 (4.3%, 1 malignant and 11 benign) were performed with GE Logiq 9, and 3 (1.1%, 3 benign) were performed with GE Logiq 7. The interclass correlation coefficient (ICC) of the two radiologists was 0.86 (95% CI, 0.76-0.92).

### Model Construction and Validation

In the training dataset, 29 nodules (19.3%) were malignant (**Table 2**). We performed univariate logistic regression analysis using age, sex, composition, echogenicity, border, margin, shape, calcification, and halo. All variables except age and shape were identified as statistically significant risk factors (**Table 2**). These risk factors were then included in the multivariable analysis. Malignancy risk factors in multivariate analysis were as follows (**Table 3**): microcalcification [odds ratio (OR) 8.37, 95% confidence interval (CI) 1.641-42.724, p = 0.011], solid (OR 1.49, 95% CI 0.391-2.566, p = 0.008), ill-defined border (OR 4.40, 95% CI 1.074-18.031, p = 0.039) and hypoechogenicity (OR 2.94, 95% CI 1.031-8.389, p = 0.044).

**Table 3:** Multivariate binary logistic regression in the training dataset.

Factor	β	B. E	Wals	P value	Exp (B)	95% CI
Microcalcification	2.125	0.831	6.533	0.011	8.374	1.641-42.724
Solid	1.479	0.555	7.105	0.008	4.388	1.479-13.016
Ill-defined border	1.482	0.719	4.242	0.039	4.401	1.074-18.031
Hypoechogenicity	1.079	0.535	4.066	0.044	2.940	1.031-8.3888
Intercept	-2.8219	0.459	-6.15	<0.0001		

Those factors with statistically significance (P<0.01) in the univariate analysis were added to the logistic analysis. CI, confidence interval.

**Figure 2:**
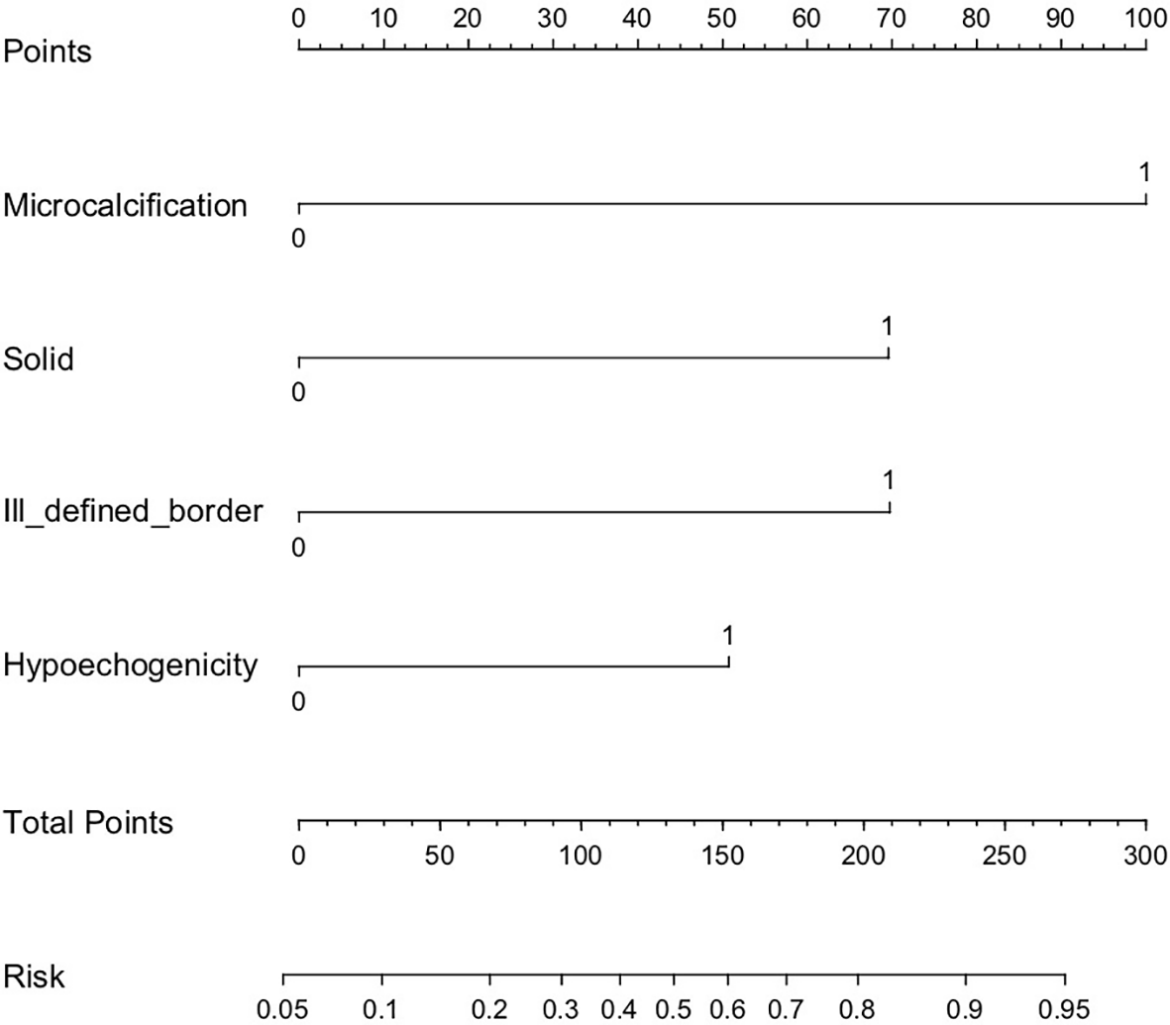
Risk stratification nomogram to predict the probability of malignancy of thyroid nodules > 4 cm. For example, a solid hypoechogenic thyroid nodule > 4 cm that shows the ultrasound feature of microcalcification, but not ill-defined border receives 221 points and a malignancy risk of 0.88 (high risk).

**Figure 3:**
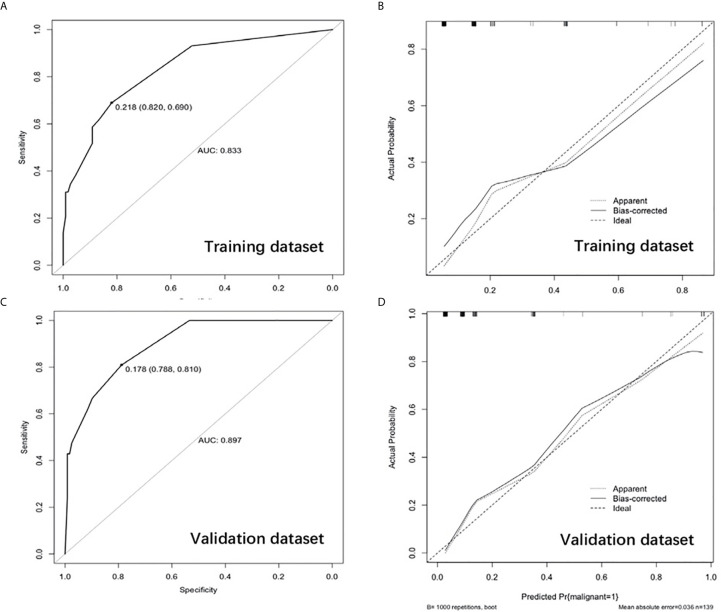
Receiver operating characteristic curve in the training dataset **(A)** and validation dataset **(C)**. Calibration curve showing nomogram-predicted malignancy compared with the actual malignancy in the training dataset **(B)** and validation dataset **(D)**.

A nomogram that integrated all four significant independent factors was constructed (**Figure 2**). The model showed a C-index of 0.833 (95% CI 0.752-0.915) for predicting malignancy in the training dataset (**Figure 3A**) and 0.897 (95% CI 0.835-0.9591) for predicting malignancy in the validation dataset (**Figure 3C**). Calibration curves for the probability of malignancy showed a good correlation between the nomogram-predicted and observed values (**Figures 3B, D**).

The risk value of each factor was calculated using the nomogram. The risk value was 0.34 for microcalcification (100 points), 0.21 for solid and ill-defined border (70 points), and 0.14 for hypoechogenicity (50 points). Using the model, all nodules were assigned to one of three risk categories (**Table 4**): nodules with more than 3 factors were classified as high risk (0.8-0.9); nodules with 2 factors or microcalcification, as intermediate risk (0.3-0.7); and nodules with 1 factor except microcalcification, as low risk (0.1-0.2).

**Table 4:** Risk stratification model for thyroid nodules > 4 cm.

Patterns	Points	Risk value	No. malignant nodules (malignancy rate, %)	
			training dataset (n = 29)	Validation dataset (n = 21)
High risk			9 (90.0)	8 (88.9)
More than 3 factors	190-290	0.8-0.9		
Intermediate risk			8 (42.1)	5 (35.7)
2 factors	120-170	0.4-0.7		
Microcalcification	100	0.34		
Low risk			12 (10.8)	8 (6.9)
Solid	70	0.21		
Ill-defined border	70	0.21		
Hypoechoic	50	0.14		

The four significant factors were microcalcification, solid, ill-defined border, and hypoechoic.

In the training dataset, the malignancy rate of thyroid nodules > 4 cm that were classified as high risk was 90.0%; intermediate risk, 42.1%; and low risk, 10.8%. The corresponding malignancy rates in the validation dataset were 88.9%, 35.7%, and 6.9% (**Table 4**). The risk stratification of the model was compared with the ACR TI-RADS (p < 0.001, **Table 5**).

**Table 5:** Comparison of malignancy risk stratification between the proposed model and ACR TI-RADS.

ACR TI-RADS	Proposed model*					
	Training dataset (n = 140)			Validation dataset (n = 139)		
	High risk	Intermediate risk	Low risk	High risk	Intermediate risk	Low risk
TR ≥4	8 (100)	9 (66.7)	9 (44.4)	9 (88.9)	11 (36.4)	5 (20.0)
TR = 3	2 (50.0)	10 (20.0)	66 (10.6)	0 (-)	3 (33.3)	69 (10.1)
TR ≤ 2	0 (-)	0 (-)	36 (2.8)	0 (-)	0 (-)	42 (0)

Values are n (%) for malignant tumors, based on pathology of surgical samples. *The risk stratification of the model > 4 cm was compared with ACR TI-RADS in training dataset and validation dataset (p < 0.001). ACR, American College Radiology; TI-RADS, Thyroid Imaging Reporting and Data System.

### Diagnostic Efficiency of the Model

Receiver operating characteristic curves demonstrated that the best cut-off value of the model was intermediate risk. In the training dataset, the model had a sensitivity of 58.8%, specificity of 84.6%, NPP of 93.7%, PPV of 34.5%, accuracy of 81.4% and AUC of 0.833 (95% CI 0.752-0.915) (**Table 6**). The AUC of the nomogram was higher than that of ACR TI-RADS (0.823, 95% CI 0.750-0.882, p = 0.011). However, the model was similar to ACR TI-RADS in sensitivity (58.8% *vs.* 58.8%, p = 0.181) and specificity (84.6% *vs.* 86.2%, p = 0.424) (**Table 6**). The AUC of the nomogram was higher than that of Kwak TI-RADS (0.817, 95% CI 0.741-0.871, p = 0.214) and ATA (0.812, 95% CI 0.735-0.874, p = 0.158). There was no significant statistical difference in the AUC value among the ACR TI-RADS, Kwak TI-RADS and ATA (p > 0.05).

**Table 6:** Diagnostic performance between the proposed model and ACR TI-RADS.

Model	Se	Sp	PPV	NPV	Ac	AUC	95% CI
Training							
model	58.8%	84.6%	34.5%	93.7%	81.4%	0.833	0.752-0.915
ACR TI-RADS	58.8%	86.2%	37.0%	93.8%	82.9%	0.823	0.750-0.882
*P*-value	0.181	0.424				0.011	
Validation							
model	61.9%	91.5%	56.5%	93.1%	87.1%	0.897	0.835-0.959
ACR-TI-RASA	57.1%	93.2%	60.0%	92.4%	87.8%	0.855	0.785-0.909
*P*-value	0.480	0.680				0.040	

ACR, American College Radiology; AUC, area under the curve; CI, confidence interval; NPV, negative predictive value; PPV, positive predictive value; Se, sensitivity; Sp, specificity; TI-RADS, Thyroid Imaging Reporting and Data System.

For the validation dataset, the model showed a sensitivity of 61.9%, specificity of 91.5%, NPV of 93.1%, PPV of 56.5%, accuracy of 87.1% and AUC of 0.897 (95% CI 0.835-0.959, **Table 6**). The AUC of the nomogram was higher than that of ACR TI-RADS (0.855, 95% CI 0.785-0.909, p = 0.040, **Table 6**). However, the model was similar to ACR TI-RADS in sensitivity (61.9% *vs.* 57.1%, p = 0.480) and specificity (91.5% *vs.* 93.2%, p = 0.680) (**Table 6**). The AUC of the nomogram was higher than that of Kwak TI-RADS (0.878, 95% CI 0.811-0.927, p = 0.445) and ATA (0.831, 95% CI 0.758-0.889, p = 0.205). There was no significant statistical difference in the AUC value among the ACR TI-RADS, Kwak TI-RADS and ATA (p > 0.05).

## Discussion

In the present study, we established a model to predict the risk of malignancy for thyroid nodules > 4 cm. This model incorporated four factors relatively easy to determine from conventional ultrasound imaging of nodules: composition, echogenicity, border, and calcification. We observed that the model achieved satisfactory diagnostic performance in both the training and validation datasets. Furthermore, the proposed model predicted malignancy better than ACR TI-RADS in both datasets, although it showed similar specificity and sensitivity as the reference standard.

The thyroid nodules > 4cm have its unique ultrasonic risk stratification. In our study, multivariate regression revealed the following independent risk factors for thyroid cancer: microcalcification (OR 8.37, 95% CI 1.641-42.724), solid (OR 1.49, 95% CI 0.391-2.566), ill-defined border (OR 4.40, 95% CI 1.074-18.031) and hypoechogenicity (OR 2.94, 95% CI 1.031-8.389). Our findings were consistent with the three suspicious malignant signs (microcalcification, solid and hypoechogenicity) in ACR TI-RADS and Kwak TI-RADS, which confirmed the effectiveness of the existing guidelines. These factors were incorporated together to develop a nomogram. This nomogram could be a useful and convenient tool in clinical practice to evaluate the malignancy risk of thyroid nodules > 4cm. The model showed a C-index of 0.833 (95% CI 0.752-0.915) for predicting malignancy in the training dataset and 0.897 (95% CI 0.835-0.9591) for predicting malignancy in the validation dataset. Calibration curve plotting demonstrated its significant predictive and discriminatory capacity in the validation cohort. Using the model, all nodules were assigned to one of three risk categories): high risk (0.8-0.9), intermediate risk (0.3-0.7) and low risk (0.1-0.2). In the training dataset, the malignancy rate of thyroid nodules > 4 cm that were classified as high risk was 90.0%; intermediate risk, 42.1%; and low risk, 10.8%. The corresponding malignancy rates in the validation dataset were 88.9%, 35.7%, and 6.9%.

The risk stratification of the model > 4cm was different with the ACR TI-RADS (p < 0.001), which indicating that the thyroid nodules > 4cm have unique characteristics of ultrasonic risk stratification.

The proposed model may be convenient to implement in the clinic. Of various factors linked to nodule malignancy, including solid, hypoechogenicity, microcalcification, taller-than-wide shape and irregular/lobulated margin (4, 6, 9), our model identified only four independent risk factors: solid, hypoechogenicity, microcalcification, and ill-defined border. This suggests that fewer ultrasound features can still provide reliable predictions of malignancy. Taller-than-wide shape is considered an insensitive but highly specific indicator of malignancy (9, 15–18), especially in sub-centimeter thyroid nodules (15, 16), and it is assigned more points (3 points) in ACR TI-RADS to reflect an association with malignancy (6). However, none of the thyroid nodules in either the training or validation datasets had taller-than-wide shape, which may indicate that shape has no diagnostic value for thyroid nodules > 4 cm. This may reflect that ultrasonography is less accurate at assessing shape, the larger the thyroid nodule is. Conversely, our study identified ill-defined border as a predictor of malignancy, similar to a previous study (19), but this factor is assigned 0 point in ACR TI-RADS. Our model and associated nomogram may be clinically easier to use than ACR TI-RADS and more accurate for thyroid nodules > 4 cm.

The model > 4cm provides a better diagnostic efficiency than the ACR TI-RADS, kwak TI-RADS and ATA.

According to Kim’s meta-analysis (20), the overall diagnostic performance of the three risk stratification systems (the ACR TI-RADS, kwak TI-RADS and ATA) of the representative society guidelines were comparable. In this study, there was no significant statistical difference in the AUC value among the ACR TI-RADS, Kwak TI-RADS and ATA in two datasets (p > 0.05). The AUC value were also no significant statistical difference (p > 0.05) among the model > 4cm, Kwak TI-RADS and ATA, similar to Shen’s study (21).

The ATA guidelines cannot cover all nodules. For example, in this study, there were two hyperechoic thyroid nodules with microcalcification which were not belong to any risk stratification of ATA guidelines. The ACR convened committees developed a set of standard terms (lexicon) for ultrasound reporting and proposed a TI-RADS based on the lexicon. All nodules can be scored in ACR TI-RADS. And ACR-TIRADS showed the lowest rate of unnecessary FNAB and highest rate of malignancy in FNAB (22–24). So, the model > 4cm was mainly compared to ACR TI-RADS. The AUC of the model > 4cm was higher than of the ACR TI-RADS, whether in training dataset or in validation dataset, and the difference has statistically significant (p < 0.05). And the model was similar to ACR TI-RADS in sensitivity (61.9% *vs.* 57.1%, p = 0.480) and specificity (91.5% *vs.* 93.2%, p = 0.680), which were higher than Ha’s study (25). This predictive model can use fewer indicators to diagnose the risk of malignant thyroid nodules larger than 4cm, and its diagnostic efficiency was consistent with that of ACR TI-RADS.

One benign thyroid nodule predicted by our model to be malignant and classified as TR5 in ACR TI-RADS illustrates the shortfalls of both systems. Ultrasonography showed that the nodule was solid, hypoechoic, and microcalcified, and that it had irregular margins and an ill-defined border. The nodule received 290 points, and malignant risk was > 0.95 according to the model. Pathology analysis of surgical samples indicated Riedel’s thyroiditis, a rare inflammatory process involving thyroid and surrounding cervical tissues that is associated with systemic fibrosis (26). The nodules associated with this condition show nonspecific ultrasound features and so are often misdiagnosed (27, 28).

This study had limitations. First, it was a retrospective study, so confounding factors could not be controlled. Second, all patients underwent thyroidectomy, which may have led to selection bias. The clinical utility of this proposed model as a preoperative decision-making tool should be explored in prospective studies.

## Conclusion

Thyroid nodules > 4 cm merit a unique ultrasonic risk stratification, and the model proposed here may outperform ACR TI-RADS. The model should be tested in large prospective studies for its ability to guide preoperative decisions.

## Data Availability Statement

The original contributions presented in the study are included in the article/supplementary material. Further inquiries can be directed to the corresponding author.

## Ethics Statement

The studies involving human participants were reviewed and approved by the Institutional Review Board of Peking Union Medical College Hospital. Written informed consent for participation was not required for this study in accordance with the national legislation and the institutional requirements.

## Author Contributions

BZ conceived and designed the study. All the other authors collected the data. XY and SZ performed the analysis. XX prepared all the figures and tables. XX, YW, and LG were major contributors in writing the manuscript. BZ edited the manuscript. All authors contributed to the article and approved the submitted version.

## Funding

This study was supported by a grant from Spatial-Temporal Mapping Analysis on Chinese Cancer Burden (2018-12M-3-003) and a grant from the National Natural Science Foundation of China (81971627).

## Conflict of Interest

The authors declare that the research was conducted in the absence of any commercial or financial relationships that could be construed as a potential conflict of interest.

## Abbreviations

ACR TI-RADS, American College Radiology Thyroid Imaging Reporting and Data System; AUC, area under the receiver operating characteristic curve; AJCC, American Joint Committee on Cancer; ATA, American Thyroid Association; TNM, Tumor, Node, Metastasis; FNAB, fine-needle aspiration biopsy; C-index, concordance index; PPV, positive predictive value; NPV, negative predictive value; CI, confidence interval.
